# A New Versatile Jig for the Calibration and Validation of Force Metrics with Instrumented Paddles in Sprint Kayaking

**DOI:** 10.3390/s24154870

**Published:** 2024-07-26

**Authors:** Hans Rosdahl, David Aitken, Mark Osborne, Jonas Willén, Johnny Nilsson

**Affiliations:** 1 The Swedish School of Sport and Health Sciences (GIH), SE-114 86 Stockholm, Sweden; david@aitkennet.net.au (D.A.); johnny.nilsson@gih.se (J.N.); 2 Paddle Australia, Silverwater, NSW 2128, Australia; mark.osborne@paddle.org.au; 3 KTH Royal Institute of Technology, Hälsovägen 11 C, SE-141 57 Huddinge, Sweden; jwi@kth.se

**Keywords:** kayaking, instrumented paddles, jig, stroke force, strain gauges, validation, elite athletes, kayakers, kinetics

## Abstract

The interest in using new technologies to obtain recordings of on-water kinetic variables for assessing the performance of elite sprint kayakers has increased over the last decades but systematic approaches are warranted to ensure the validity and reliability of these measures. This study has an innovative approach, and the aim was to develop a new versatile jig including reference force sensors for both the calibration and validation of mutual static and dynamic stroke forces as measured with instrumented paddles at the high force levels used in elite sprint kayaking. Methods: A jig was constructed using a modified gym weight stack and a frame consisting of aluminum profiles permitting a fastening of custom-made kayak paddle shaft and blade support devices with certified force transducers combined with a data acquisition system to record blade and hand forces during static (constant load) and dynamic conditions (by paddle stroke simulation). A linear motion path incorporating a ball-bearing equipped carriage with sensors for the measurement of vertical distance and horizontal displacement was attached to the frame for recordings of various position measures on the paddle. The jig design with all components is extensively described to permit replication. The procedures for assessing the accuracy of the jig force instrumentation are reported, and with one brand of instrumented paddle used as an example, methods are described for force calibration and validation during static and dynamic conditions. Results: The results illustrate that the measured force with the jig instrumentation was similar to the applied force, calculated from the applied accurate mass (within a −1.4 to 1.8% difference) and similar to the force as calculated from the applied mass with the weight stack (within a −0.57 to 1.16% difference). The jig was suitable for the calibration and validation of forces in a range relevant for elite sprint kayaking under both static and dynamic conditions. During static conditions with a force direction equal to the calibration conditions and a force range from 98 to 590 N, all values for the instrumented paddle were within a −3.4 to 3.0% difference from the jig sensor values and 28 of 36 values were within ±2%. During dynamic conditions with paddle stroke simulations at 60 and 100 strokes/min and a target peak force of 400 N, the common force variables as measured by the instrumented paddle were not significantly different from the same measures by the jig (values at 100 strokes/min: peak force; 406.9 ± 18.4 vs. 401.9 ± 17.2 N, mean force; 212.8 ± 15.4 vs. 212.0 ± 14.4 N, time to peak force; 0.17 ± 0.02 vs. 0.18 ± 0.02 s, force impulse; 90.8 ± 11.2 vs. 90.5 ± 10.8 Ns, impulse duration; 0.43 ± 0.03 vs. 0.43 ± 0.03 s). Conclusion: A novel jig with several new functions is presented that enables the calibration and validation of force measurements with instrumented paddles by providing standardized conditions for calibration and force validation during both static and dynamic conditions in a force range relevant to elite sprint kayaking.

## 1. Introduction

**Figure 1:**
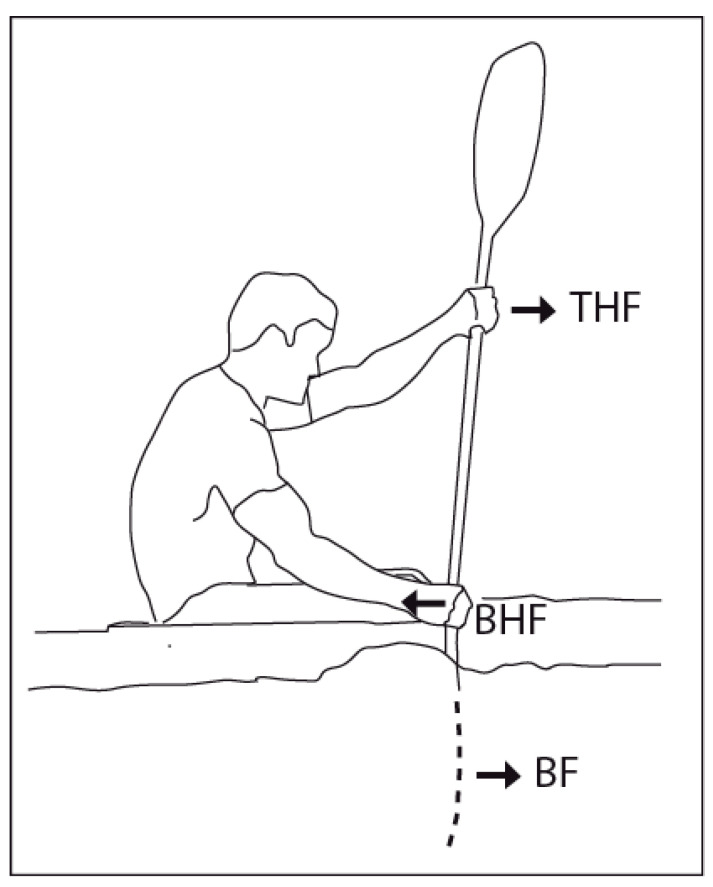
Forces on the paddle shaft and blade in kayaking. A stroke on the right side is shown with the right hand closest to water (i.e., the bottom hand creating an action force, “Bottom Hand Force”, BHF) and the left hand creating an action force (the “Top Hand Force” THF in a force couple with BHF). During a stroke on the left side, the right hand creates THF and the left BHF. The kayak movement in the forward direction is generated by the action forces (BHF and THF) resulting in a reaction force between the blade and the water, “Blade Force” (BF).

The sport discipline of sprint kayaking is characterized by male and female athletes competing in either single (K1), double (K2), or four (K4)-person boats, over race distances of 200, 500, 1000, and 5000 m. High levels of technical skill and physical capacity are required to succeed at international competitions. Determinants to performance can be considered as the ability of the athlete to utilize their metabolic and neuromuscular capacity to produce optimal power during each stroke [1]. Depending upon the race distance and boat class, the athlete may be required to produce between 80 and 360 individual paddle strokes or 40 and 180 stroke cycles. Given the highly cyclical nature and the importance of force application for the forward kayak movement, it has become of great interest to record the forces transferred through the blade via both hands at the elite level (Figure 1). In accordance, the assessment of stroke kinetics using strain gauges, or strain gauge-based sensors, attached to the shaft [2,3,4,5,6,7] or embedded in the shaft [8,9] has become more common for measures both during kayaking on water and in the laboratory using a kayak ergometer [10,11]. In addition, force sensors placed on the paddle have recently been used in a study on the physics of kayaking to link the propulsion force to the change in kayak velocity [12]. As Tanner and Gore note, a prerequisite to accuracy is the calibration of equipment preferably utilizing the first principles of time, distances, or mass [13]. In line with this, usually, all strain gauge-based sensors require calibration by establishing the relationship between the transducer output and a known applied mass to set the scaling for the measurement of force. The calibration and validation of instrumented paddles is an essential step to obtain accurate and reliable data which is of utmost importance for athletes and coaches.

One example of an instrumented paddle that requires calibration using the first principle of mass is the Kayak Power Meter generation 2.1 developed by One Giant Leap (OGL, One Giant Leap, Nelson, New Zealand, see Section 2). This device utilizes strain gauges attached to the internal sections of the paddle shaft to measure bending in the shaft proportional to forces applied by the top (push) and bottom (pull) hands. The OGL Power Meter can record and store the applied forces at 100 Hz in an internal memory for subsequent downloading and analysis. 

Despite the increasing development of strain gauge-based instrumented paddles with associated data acquisition systems, the equipment and principles for calibration and validation have received less attention. The commonly used principles, procedures and jigs for calibrating instrumented paddles for measurements of stroke forces were described more than 30 years ago by Aitken and Neal [7] and are limited to static calibration (constant load) with load force applied by manually suspending masses on the shaft, and hence, do not permit a dynamic force application (changing loads such as those simulating paddle strokes at different rates). The orientation of load force on the shaft was only orthogonal relative to the face of the blade and did not allow for a rotation of the blade for the validation of forces not orthogonal relative to the face of the blade which is present partly during paddling at ecological conditions [14]. In addition, the jig did not include any sensors for reference force measures and did not allow any additional devices to be attached. 

Following this, one study has recently presented a new approach using a test bench with a lever arm, joint mechanism and reference force sensor to provide a pressure force at both static and dynamic conditions at a 1 × 1 cm^2^ support target on the shaft [15]. However, it was designed specifically for the characterization and calibration of piezoresistive sensors and its versatility for commonly used strain gauged-based instrumented paddles is unclear at the present time. Also, since the maximal force expected to occur during kayaking on water was below 375 N, the jig was limited to a maximal force of 500 N and is therefore not covering the entire range of peak forces expected with male elite kayakers (the requirements are discussed in the Discussion section). Furthermore, it did not allow for a rotation of the blade for the validation of forces not orthogonal relative to the face of the blade.

This study has an innovative approach, and the aim was to develop a new versatile jig including reference force sensors for both the calibration and validation of mutual static and dynamic stroke forces as measured with instrumented paddles at the high force levels used in elite sprint kayaking.

## 2. Materials and Methods

### 2.1. Outline

The first part of this section includes an extensive description of the jig design with all components described to permit replication (Section 2.1.1, Section 2.1.2, Section 2.1.3, Section 2.1.4 and Section 2.1.5). A photo of the jig is in the Figure A5. The second part includes a description of the procedures for assessing the accuracy of the jig force instrumentation (Section 2.1.6 and Section 2.1.7). The third part includes examples of using the jig for the calibration and validation of forces recorded from an instrumented paddle. The procedure for calibration is described in Section 2.1.8, followed by a procedure for the validation of the force measurements during static conditions with the orientation of load force that is orthogonal relative to the face of the blade (Section 2.1.9) and a procedure with load force that is not orthogonal relative to the face of the blade (Section 2.1.10) followed by a procedure for the validation of force measures during dynamic loading (paddle stroke simulation, similar to kayaking on water, Section 2.1.11). 

#### 2.1.1. General Jig Frame and Weight Stack

**Figure 2:**
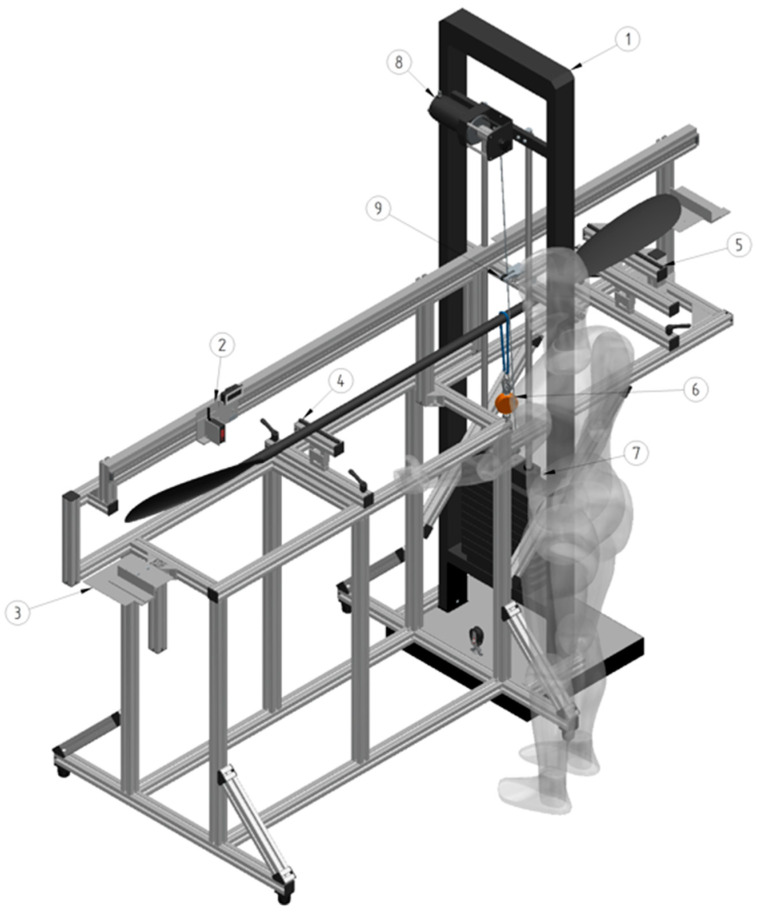
General jig frame and weight stack with an instrumented paddle operated by a person in front of the jig. The major parts of the jig consist of (1) a frame for the weight stack, (2) a linear motion path with sensors to measure displacement and positions, (3) plates to define the start and the end of paddle length measures, (4) a shaft support with a force sensor beneath it, (5) a blade support with a force sensor beneath it, (6) a force sensor and a load cable, (7) a pin-loaded weight stack (12 × 5 kg), (8) an electrical winch and (9) a load cable steering device.

**Figure A1:**
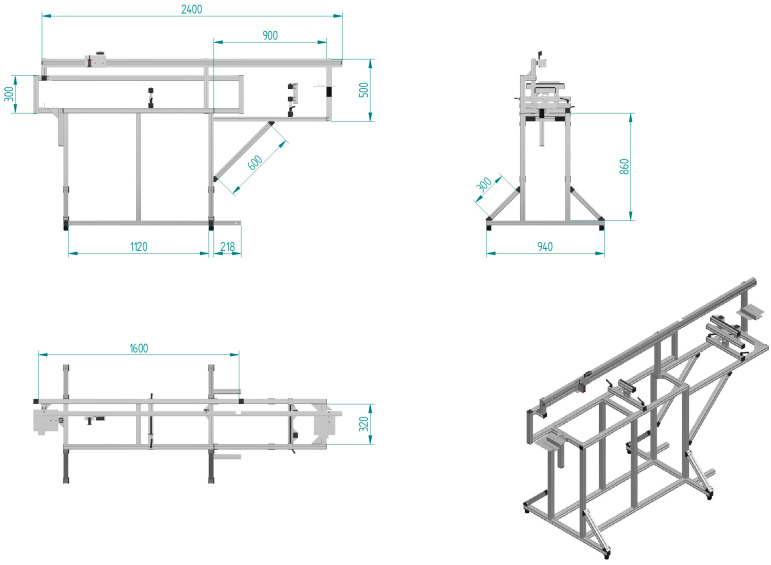
General jig frame with basic measures (mm).

**Figure A2:**
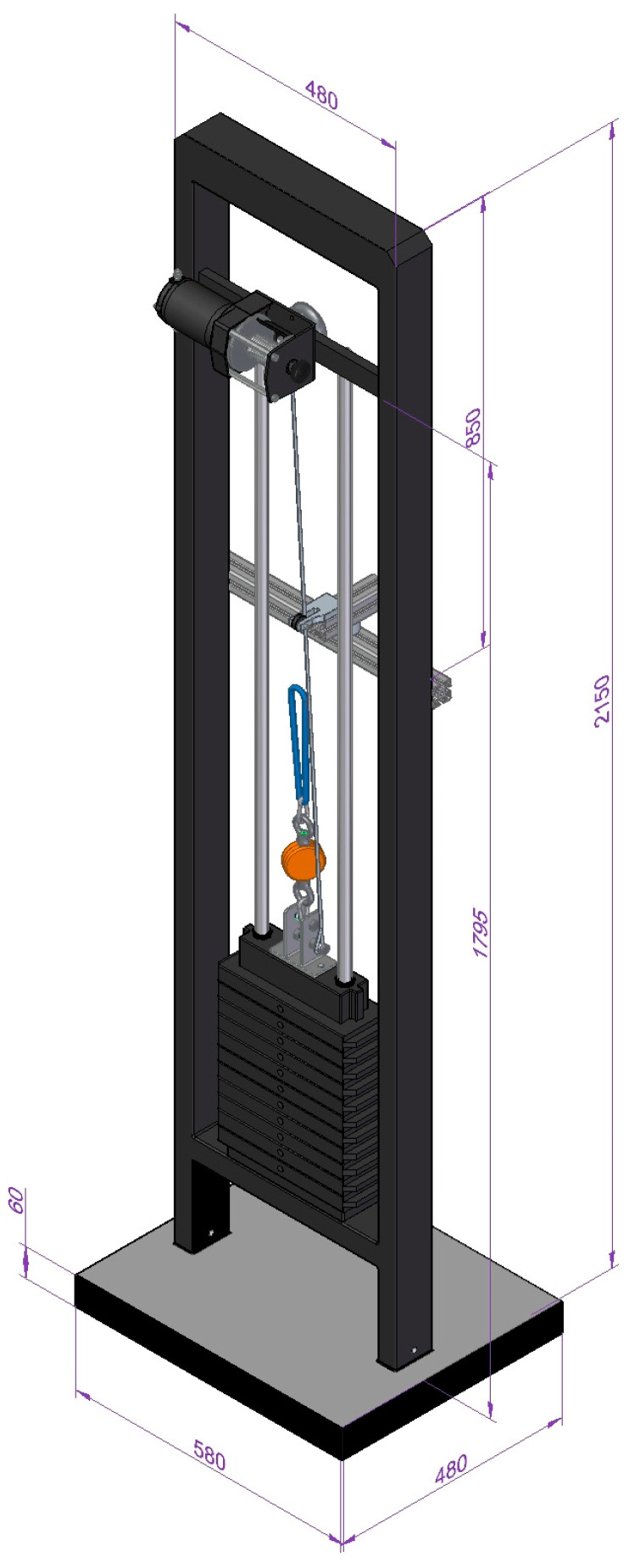
Weight stack with basic measures (mm).

The basic frame of the jig (Figure 2) consists of commercially available aluminum components (item Industrietechnik, GmbH, Solingen, Germany). Aluminum profiles with open grooves (Profile system 8, 40 × 40 mm, Art No 0.0.026.03) permit a versatile fastening of various panel elements and are, together with additional panel elements, interconnected with standard fastening set 8 (Art No 0.0.026.07), angle bracket set 8, 40 × 40 mm (Art No 0.0.411.15), angle bracket set 8, 80 × 80 mm (Art No 0.0.411.32), angle element 8 T1-40 (Art No 0.0.388.00) and T-Slot Nut 8 (Art No 0.0.026.18) with M8 thread screws of various lengths. For horizontal level adjustments, adjustable feet (foot 8 PA, Art No 0.0.196.64) are attached underneath the frame (see Figure A1). A separate frame with a pin-loaded weight stack is attached to the basic jig frame using bolts (M8) at the bottom and mid sections of the jig (see Figure A2). To raise and lower the weights, a battery-powered electrical winch (Biltema 15-510, 12 V max load 907 kg, Helsingborg, Sweden) is attached at the upper part of the frame. An aluminum profile with a block is mounted on the weight stack frame to steer the load cable in line with the weights. A photo of the jig with acting forces illustrated can be found in the Figure A5. 

#### 2.1.2. Linear Motion Path with Display and Sensors for Measurement of Horizontal and Vertical Positions

**Figure 3:**
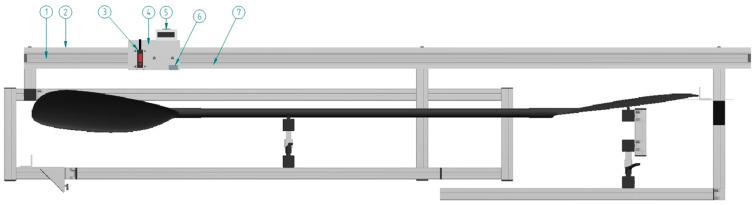
Linear motion path with a ball-bearing carriage seen from the front. The linear motion path consists of the (1) motion path, (2) an aluminum L-profile, (3) a high precision laser for vertical displacement measures, (4) a plate on the ball-bearing carriage, (5) a display for horizontal position measures, (6) a magnetic sensor for horizontal position measures and (7) a magnetic strip for horizontal position measures.

To enable accurate measurements of total paddle length, hand positions, other spots and the alignment control of the shaft, the linear motion path with display and sensors for the measurement of horizontal and vertical positions was developed. A commercially available aluminum linear motion path (Accuride DA0115-0240RC, length 2400 mm, height 40 mm, Accuride International Inc., Santa Fe Springs, CA, USA) is attached, together with a 70 × 30 × 3 mm aluminum L profile for stabilization, to the general frame to permit linear, horizontal and vertical position measurements (Figure 3). A recirculating ball-bearing carriage, with a length of 111 mm, height of 40 mm (DS0115-CASSRC), customized with an aluminum plate to carry the sensors for reference point displacement measurements, is attached to the motion path. The linear horizontal reference position measurements are performed with a magnetic measuring system (Elesa LTD, Lincolnshire, UK) consisting of a plate with a display and a keyboard (MPI-R10), a magnetic sensor (FC-MPI), and a magnetic band (M-Band-10-25) attached along the linear motion path. According to the manufacturer’s technical specifications, the linear precision of this system is ±0.03 mm and repeatability is 0.0002 × L mm (L = the value measured in mm). The vertical position is measured by a high-precision laser (HL-G112-A-C5, Panasonic Industry Co., Ltd., Osaka, Japan), with the additional function of being used to align the paddle shaft on the jig with the linear motion path. According to manufacturer specifications, the measuring range of the laser is ±60 mm, the resolution is 8 µm and the linearity is ±0.1% F.S.O. 

**Figure A3:**
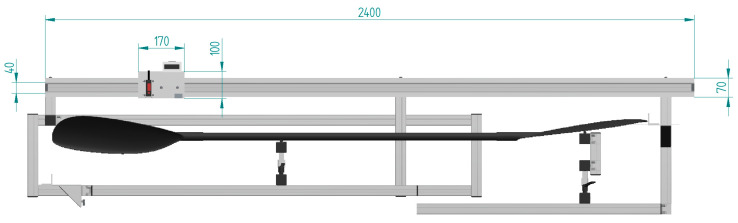
Linear motion path with basic measures (mm).

To establish a precise start and stop during measurements of total paddle length, using a combination of the laser and magnetic measuring system, custom-made plates (Left 200 × 170 mm, Right 160 × 160 mm) with reference lines are connected at each side of the jig frame. The plates are mounted on top of vertically adjustable aluminum profiles. Separate and freely movable L-profile plates (40 × 40 × 3 mm) are used to align the edge of the paddle blade and the reference lines. See Figure A3 for the measure details of the linear motion path.

#### 2.1.3. Adjustable Support Devices for Shaft and Blade Support

**Figure 4:**
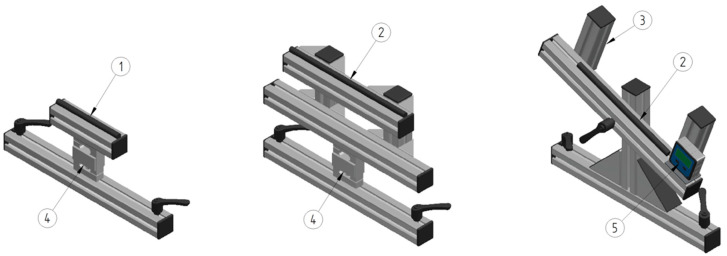
Adjustable support devices for the paddle shaft and blade. The adjustable support devices consist of (1) a shaft support, (2) a horizontal blade support, (3) a vertical blade support, (4) a force sensor, and (5) a digital protractor.

A shaft support at the top hand position consists of a bottom aluminum profile (L = 400 mm) and a top profile (L = 160 mm) with the force sensor mounted in between. To keep the shaft stable at the correct position, an 11 × 150 mm glue stick with a custom-made circular cut at mid position, (Cotech, Art No 20-4099, Clas Ohlson AB, Insjön, Sweden) is placed in the groove on the top profile. This device has a fixed vertical position but can be shifted horizontally and fixed at the preferred position (Figure 4, left).

Shaft support at the blade position consists of a bottom aluminum profile (L = 400 mm), a middle profile (L = 400 mm) with a force sensor mounted in between, and a top profile with a glue stick (see above) placed in the groove to ensure feasible support at the edges of the paddle blade. The top profile is connected to the middle profile with two vertical profiles (L = 150 mm). Hence, this device can be adjusted vertically and horizontally (Figure 4 middle).

**Figure A4:**
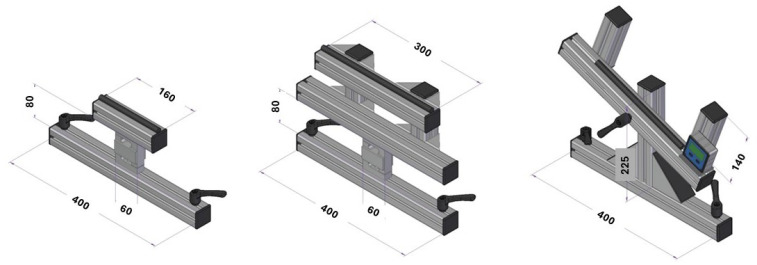
Adjustable support devices with basic measures (mm).

Another shaft support at the blade position, made to be rotatable to study force directions other than those perpendicular to the shaft, consists of a bottom profile (L = 400 mm) connected with two angle brackets (set 8, 80 × 80 Art No 0.0.411.32) to a vertical profile (L = 230 mm) and a top aluminum profile (L = 400 mm) with two vertical adjustable profiles (L = 120 mm) to keep the blade in a fixed position. A rotation from −20 to +20 degrees is viable through a bolt in the middle of the top profile. Thus, the paddle blade can be rotated and adjusted both vertically and horizontally. A digital protractor (Holzkraft, item no. 5910094, Stürmer Maschinen GmbH, Hallstadt, Germany) is attached to the top profile to monitor the angle of the paddle blade when rotated (Figure 4 right). See Figure A4 for details of the adjustable support devices.

#### 2.1.4. Setup for Force Measurements during Application of Dynamic Forces and Stroke Simulation

**Figure 5:**
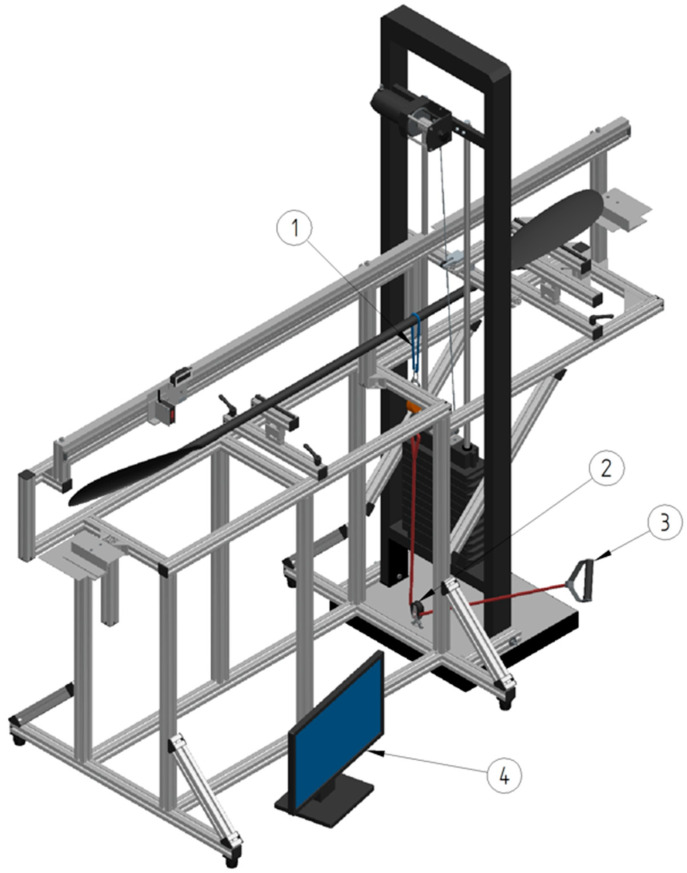
Setup for the application of dynamic forces and simulated kayak strokes. For the application of dynamic forces, i.e., paddle stroke simulation, the setup of the jig is supplemented with (1) a load sling and force sensor at the bottom hand position, (2) a block with ball bearings, (3) a hand grip, and (4) a monitor for real-time feedback of force production.

The setup for the dynamic application of force is shown in Figure 5. The paddle is positioned horizontally between supports underneath the top hand center position (paddle left side) and at the middle of the right blade. Beneath each support is a force transducer positioned (see Figure 4) to record forces in the vertical direction. At the center of the bottom hand position is a cord attached to the shaft with a sling. The cord is vertically aligned and connected to a force transducer (see below) and further to a ball-bearing equipped block/pulley. At the end of the cord, a force can be applied manually (via the attached handle) or by means of an actuator/step motor, if available. The forces from calibration jig sensors are recorded simultaneously. When the force is applied to the bottom hand center position manually, a metronome may be used to help maintaining the “paddler’s stroke rate”. 

#### 2.1.5. The Data Acquisition System

The data acquisition system (DAQ) used with the jig contains three force sensors, one fastened between the jig frame and support device for the shaft, and one fastened between the frame and blade support. Both are pre-calibrated and delivered with a calibration certificate approving 300 kg of maximal capacity with linearity, a total error < 0.02% Rated Load, non-repeatability 0.02% Rated Load and zero return < 0.017% load, the temperature effect on zero balance < 0.0040% R.O./Deg C, the temperature effect on span < 0.0012% load/Deg C, (Tedea-Huntleigh Electronics. CO. LTD, Model 616, Beijing, China, acquired via BLH Nobel, Karlskoga, Sweden). Another pre-calibrated force sensor is attached between the weight stack and the rope around the paddle shaft (KRG-4 T10, 2 kN, BLH Nobel Karlskoga, Sweden). For transducer excitation, bridge output signal amplification, and visual display, all force sensors are connected to a G4 multi-channel force instrument (BLH Nobel, Karlskoga, Sweden) set up to measure force at 300 Hz with analog output. The analog to digital conversion, down-sampling to 100 Hz, data filtering, raw data storage, and file export to Excel format is accomplished with the CED Micro 1401-4 laboratory interface and the CED Spike 2 software for Windows 10 (Cambridge Electronic Design Limited, Cambridge, UK). 

#### 2.1.6. Investigation of the Accuracy of Force Sensor Measures with the Jig

To investigate the accuracy of the force measures with the jig sensors, calibration masses with verified accuracy were applied at the center of the support devices for the top hand and the blade. For the bottom hand transducer, a connector to the transducer was attached to the jig weight stack, thus creating known forces in the perpendicular direction. Six 10 kg calibration masses were applied in a stepwise order, twice. The first series (A) was from 0 to 60 kg and the second (B) was from 60 to 0 kg. The unloaded periods in both series were used to check for hysteresis. Data were collected continuously from each jig force sensor by the DAQ described above for 120–130 s with each load applied until steady state (approximately within 15–20 s). A two seconds average was used once a steady state had been achieved, to establish the average value for each load. The accuracy of the calibration masses was verified with a high precision balance (CAS 30 kg PB, CAS Corporation, Yangju-si, Republic of Korea) and these recordings were used to convert mass (kg) to force (N). The results of the force as measured by the jig force sensors and the DAQ are provided in Table 1. 

#### 2.1.7. Investigation of Accuracy of Force Generated with Weight Stack Loading

Forces generated by the weight stack were investigated by connecting the bottom hand force sensor between the weight stack and the steel cable of the electric motor. With this setup, all weights were applied three times consecutively for 8–10 s and in a stepwise order from 10 to 60 kg with 10 kg in each step. The results provided in Table 2 are 2 s averages for each load and the average of three values at each load. 

#### 2.1.8. Jig Operation during Calibration of an Instrumented Paddle

This section outlines how the jig can be used for the calibration of instrumented paddles by applying accurate masses at the bottom hand position with stable support at the top hand and blade positions. It is also possible to include force recordings with the jig force sensors and DAQ in this procedure; however, since it is not required, it is not included here. However, it can be found below in 2.1.9. Prior to calibration, the instrumented paddle, One Giant Leap generation 2.1, was assembled, configured, and calibrated in accordance with instructions from the manufacturer (https://support.onegiantleap.co.nz/v/2.1/ (accessed on 17 February 2022). In brief, the procedure was initiated by paddle blade assembly and followed by a configuration step including downloading recent firmware (v0.2.15) and measuring the blade tip-to-blade tip distance, blade tip-to-hand distances, and blade tip-to-datum distance with the jig horizontal reference position measuring system. The distance between hands was obtained through the blade tip-to-hand distances marked on the shaft by adhesive plastic tape (the distance between hands equals the blade-to-tip distances subtracted from the tip-to-tip distance) and was kept the same as on the athlete’s regular paddle. The results of these measures were entered into the configuration page in the OGL Web Bluetooth App. 

**Figure A5:**
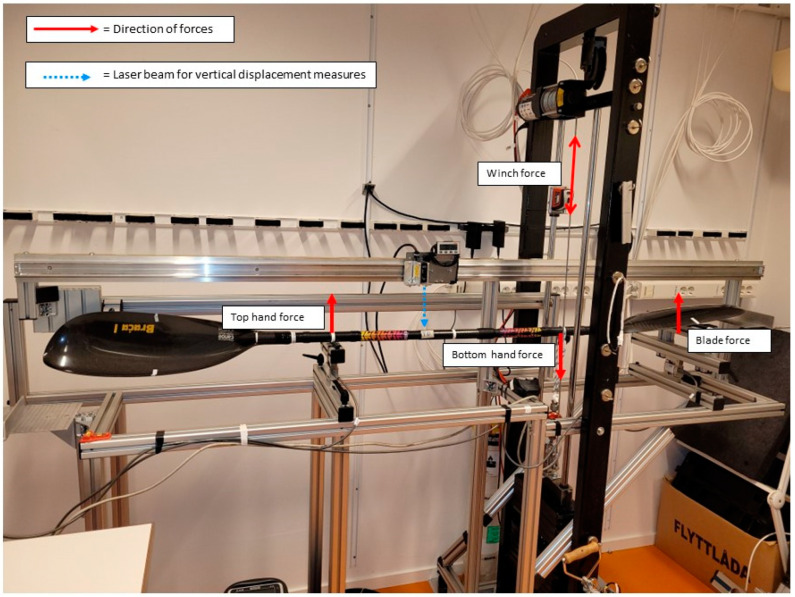
Photo of the jig in the real world.

Calibration was initiated by entering information about the applied masses and measures of the tip-to-close support, tip-to-far support, tip-to-applied load, tip-to-datum (marked position), datum-to-blade gauge, datum-to-center gauge, datum-to-extra gauge and datum-to-datum into an Excel spreadsheet for calibration. Thereafter, the total paddle length was verified, and the paddle twist angle was adjusted to 90 degrees relative to the mid-center scale of the paddle. Following this, the paddle was placed in the jig with the bottom hand position aligned with the force direction of the weight stack. The jig blade support device was adjusted to the center of the blade and the top hand support device was adjusted to the mark on the shaft for the top hand position (see Figure 2 and Figure A5). Subsequently, the calibration page in the OGL Web Bluetooth App, connected online with the paddle, was selected and a zero offset was obtained by selecting “zero offset” and the observed values were entered into the calibration spreadsheet. Following this, the first calibration mass was applied, and the live output values of angles and A, B, and C gauge channels were read and entered into the calibration spreadsheet for scale factor calculation. This procedure was repeated with all four masses (10, 20, 30, and 40 kg) applied. Finally, the scale factors were retrieved from the calibration spreadsheet and entered into the calibration page of the OGL Web Bluetooth App online connected to the instrumented paddle. 

#### 2.1.9. Jig Operation, Including Jig Sensor Force Recordings with the DAQ for Validation of Force Recordings during Static Conditions and the Orientation of Load Force That Is Orthogonal Relative to the Face of the Blade

This section outlines how the jig can be used for the validation of forces measured by instrumented paddles after they have been calibrated. The procedure is similar to calibration, applying accurate masses at the bottom hand position with stable support at the top hand and blade positions, but in this example, masses of 50 and 60 kg are also applied to test linearity at forces expected from elite athletes during kayaking on water at ecological conditions (highest mass applied in calibration is 40 kg). This example also includes force recordings by the jig sensors and DAQ as a reference to provide the opportunity to verify the forces measured by the instrumented paddle at the precise positions of the top hand, bottom hand and center of the blade.

This procedure was initiated by adjusting the paddle to its specific total length and blade twist angle and then placed in the jig with the bottom hand position aligned above the force direction of the weight stack. Thereafter, the jig blade support was placed at the center of the blade and the top hand support at the mark on the shaft for the top hand position. The paddle-specific settings such as force sensor gain, etc. (see above), were checked and the current offset values were set via the OGL Web Bluetooth App. Offset for the jig force measuring system was set via the front panel of the G4 instrument. To standardize data collection processes, each stage of the protocol was voice-recorded on a cell phone and re-played during each subsequent collection. Data collection was started simultaneously with the instrumented paddle via the OGL Web Bluetooth App and High-Speed Data (HSD, 100 Hz) recording page, and the Spike 2 software for the jig force measuring system. Each collection period was completed in 2:08 min and was initiated and ended with an 8 s period where mass was unloaded for baseline recording. Each mass (see Table 2) was applied for 8 s, with 12 s in between for changing mass. The procedure was first used on the right side of the instrumented paddle (right blade supported and shaft loaded at the right bottom position) and then repeated for the left side. Data recorded within the instrumented paddle were downloaded wirelessly using an ANT+ Garmin USB stick and ANTFS PC Host software https://www.thisisant.com/resources/ant-fs-pc-tools (accessed on 15 December 2020). Each file, being in fit format, was opened, viewed, and exported to CSV format with specific software https://fit.onegiantleap.co.nz/ (accessed on 17 February 2022) imported into Excel, and saved in Excel format. Data from the jig force sensors were recorded and processed in the first step using the Spike 2 software (see above) and exported in Excel format. Afterward, all data from the instrumented paddle and the jig force sensors were opened in Excel, aligned, and averaged in 3 s periods, 3–5 s (baseline), 12–14 s (10 kg), 32–34 s (20 kg), 52–54 s (30 kg), 72–74 s (40 kg), 92–94 s (50 kg), 112–114 s (60 kg) and 122–124 s (baseline), and further processed. The results from these measures are provided in Table 3 below.

#### 2.1.10. Jig Operation for the Validation of Force Measurements during Static Conditions and the Orientation of Load Force That Is Not Orthogonal Relative to the Face of the Blade 

This section outlines how the jig can be used for the validation of forces measured by instrumented paddles with forces applied in directions different from the calibration situation, i.e., with the orientation of force that is not orthogonal relative to the face of the blade, achieved by changing the angle of the blade support, resulting in a rotation of the shaft and varied force directions at the top and bottom hand positions on the shaft. This type of validation is relevant since the forces during kayaking on water are not orthogonal relative to the face of the paddle blade at sub-phases of the stroke. 

A rotatable device for blade support was assembled and replaced with a vertical support device (Figure 4) to allow varied force directions in relation to the paddle shaft. Subsequently, an instrumented paddle was placed and configured in accordance with the description in Section 2.1.9 above. Likewise, the same loading protocol, procedure for the start and stop of HSD collection, and further data processing were used. In the first data collection, the right side of the paddle was loaded with the force direction perpendicular to the shaft, and in the following, the force direction changed, respectively, to be +10, +20, −10, and −20 degrees from perpendicular. The same procedure was then repeated for the left paddle side. Thereafter, all data recorded in the instrumented paddle and the jig DAQ were downloaded and further processed as described in Section 2.1.9 above. The results from these measures are provided in Table 4 below.

#### 2.1.11. Jig Operation for Validation of Force Measurements during Dynamic Conditions with Paddle Stroke Simulation

This section outlines how the jig can be used for the validation of forces measured by instrumented paddles with forces applied during dynamic conditions such as kayaking on water with rapid changes in force over time during each stroke. It also includes force recordings by the jig sensors as a reference being necessary to verify the forces measured by the instrumented paddle.

To enable dynamic force application in a setup as described in Section 2.1.4 above, the cord was vertically aligned to the ball-bearing equipped block and serially connected to the force sensor. The support devices for the top hand and the paddle blade were moved left (Figure 5) to match the correct positions of the top hand and the blade center. Following this, the paddle was placed and configured in accordance with the description in Section 2.1.9 above and the data collection followed the same procedure. 

Each side of the instrumented paddle was loaded with six different force levels at three different stroke rates and data collection was separated into three separate sequences on each side. The stroke rate was kept constant using a metronome set at either 60, 80, or 100 strokes/min. At each rate, 10 strokes were applied at peak forces of 100, 200, 300, 400, 500, and 600 N with a 5-s pause between each peak force level. All data recorded in the instrumented paddle and the jig DAQ were thereafter downloaded and further processed as described in Section 2.1.9 above. 

**Figure 6:**
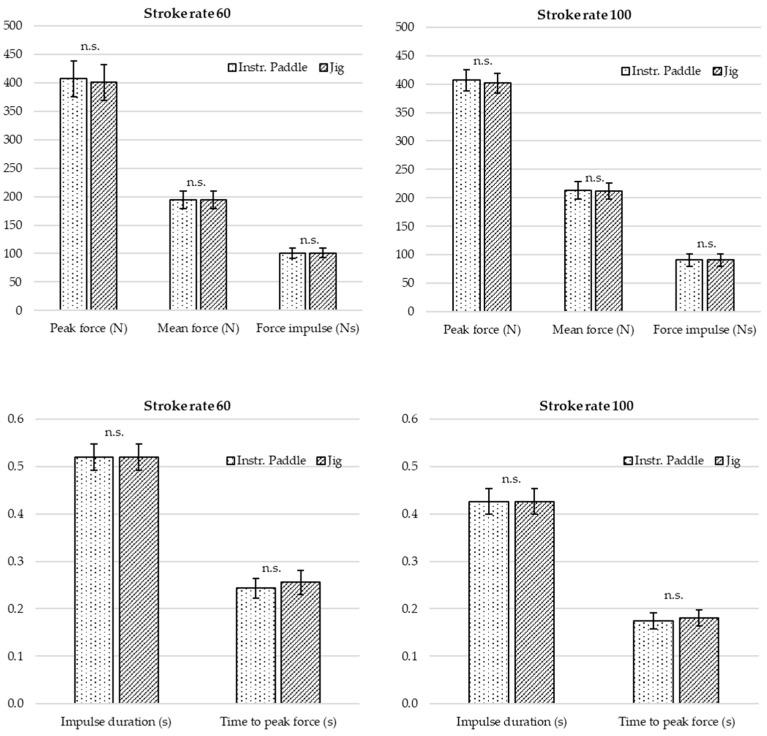
Force and time-related variables with dynamic force applied at 60 and 100 strokes/min. The calculated variables as measured by the instrumented paddle were not significantly different (n.s.) from the jig values.

**Figure A6:**
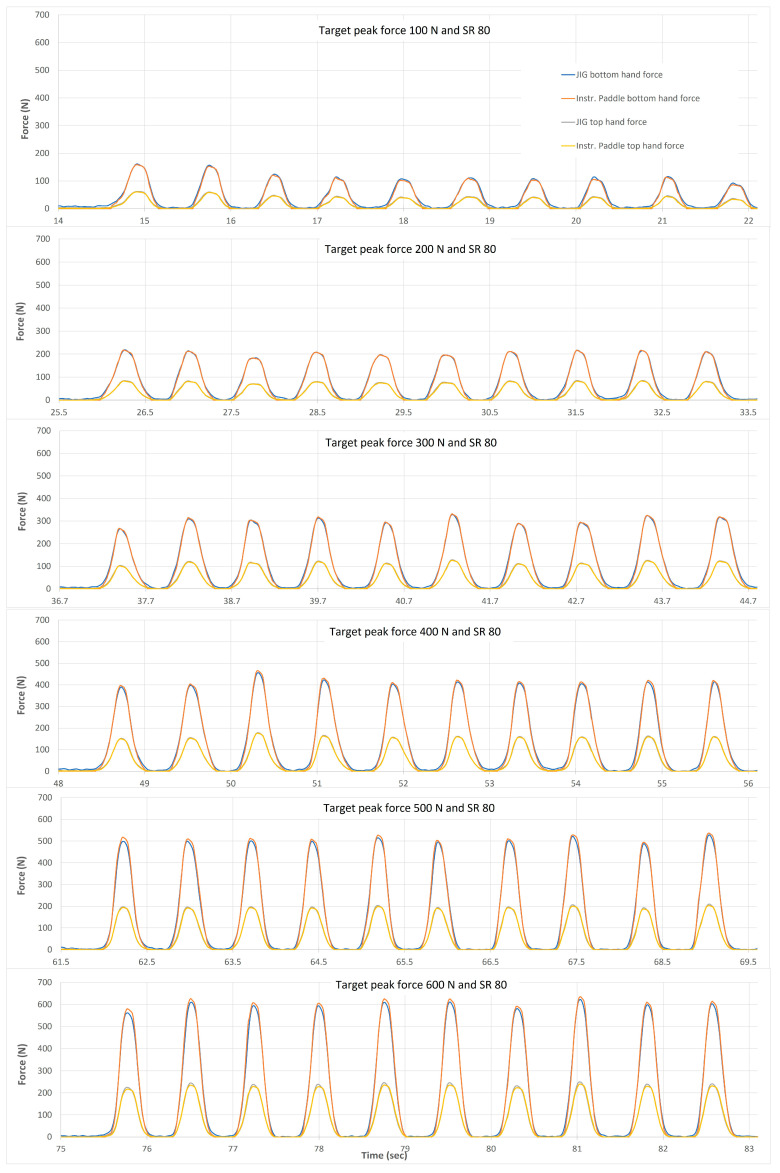
Force vs. time at the target stroke rate of 80/min and target peak forces 100–600 N.

All 10 strokes from each force level were used for further data processing. The mean and standard deviation of all sets of 10 strokes were tabulated and then depicted in Figure 6. The results of these measures with data for each stroke are available in Appendix A Table A1 and graphs of force concerning time from additional recordings with target forces applied from 100 to 600 N at 80 strokes/min are available in Figure A6. Differences between measures made by the jig sensors and the instrumented paddle were evaluated by two-tailed independent *t*-tests for equal variance. Before analysis, an equal variance was secured by F-tests (F-values ranged from 0.52 to 1.00).

## 3. Results

### 3.1. Accuracy of Force Sensor Measures in the Jig

The data in Table 1 reveal that the measured force with the jig sensors was close to the force as calculated from the applied mass. All values were within a −1.4 to 1.8% difference and half of the values were within a −0.39 to 0.46% difference. 

### 3.2. Accuracy of Forces with Weight Stack Loading

The results in Table 2 illustrate that the measured force with the jig sensors was close to the force as calculated from the applied mass with the weight stack. All values were within a −0.57 to 1.16% difference. 

### 3.3. Validation of Instrumented Paddle Forces with the Orientation of Load Force That Is Orthogonal Relative to the Face of the Blade 

The results in Table 3 provide an example of how forces measured with the sensors in the jig at the top and bottom hand positions on a shaft and the blade position can be used to validate forces recorded by an instrumented paddle. Since the blade force was measured with the force sensors in the jig only, the blade force given for the instrumented paddle shaft represents calculated values by subtracting the top from the bottom hand force. All force values from the jig were measured concomitantly with the instrumented paddle during the application of force. In this example, all values for the instrumented paddle were within a −3.4 to 3.0% difference from the jig sensor values and 28 of 36 values were within ±2%. 

### 3.4. Validation of Instrumented Paddle Forces with the Force Direction That Is Not Orthogonal Relative to the Face of the Blade

Table 4 shows how forces at the hand positions, as measured by the jig sensors in the vertical direction, may be used to validate forces recorded with an instrumented paddle when the force direction is changed by ±10 and ±20 degrees from the orthogonal direction relative to the face of the blade. In this example, all values for the instrumented paddle were within a −2.9 to 4.1% difference from the jig sensor values. 

### 3.5. Validation of Instrumented Paddle Forces during Dynamic Force Application, i.e., Paddle-Stroke Simulation

The results presented in Figure 6 show examples of common variables, such as peak force, mean force, and force impulse, that can be calculated from dynamic force application in the jig to mimic paddle strokes during kayaking on water. These variables were calculated both from the force curves of the sensors in the jig and the instrumented paddle shafts (OGL KPM gen 2.1). A target peak force of 400 N was applied manually at stroke rates of 60 and 100/min and each variable represents a mean ± SD of 10 strokes. 

In Figure 6, common time-related variables such as force impulse duration and time-to-peak force are shown. In this example, the calculated variables as measured by the instrumented paddle were not significantly different from the jig values. Data for each stroke and *p*-values from the statistical evaluation are available in Table A1. Graphs of force with respect to time from additional recordings with target forces applied from 100 to 600 N at 80 strokes/min are available in Figure A6. 

## 4. Discussion

This study is an innovative approach to develop a new versatile jig including reference force sensors for both the calibration and validation of mutual static and dynamic stroke forces as measured with instrumented paddles at the high force levels used in elite sprint kayaking. To the best of our knowledge, this is the first time a versatile jig has been extensively described allowing the first principles to be used for both calibration and validation of force metrics with instrumented paddles during both static and dynamic conditions in the high force range applicable to elite-level kayaking. 

### 4.1. Investigation of the Accuracy of Force Sensor Measures with the Jig Sensors and the Accuracy of Force Generated with Weight Stack Loading

Different from previous calibration jigs, the frame of the jig in the present study permitted the attachment of force sensors placed in adjustable support devices for the paddle shaft and blade to measure forces at all necessary positions, i.e., the top hand (push), bottom hand (pull) and blade positions (forward driving force for the kayak). Although certified force sensors were used for this purpose, we investigated the accuracy of them by loading the support devices with known masses to find out if they were appropriate to serve as reference measures for the validation of forces as measured by instrumented paddles in our setup. Founded in our results showing that all force measures by the jig sensors were within a −1.4 to 1.8% difference from the applied force over the whole range, we find them appropriate to fulfill the purpose to be used for the validation of forces as measured by instrumented paddles. In addition, because the load during actual measurements with instrumented paddles is generated by the weight stack, we investigated the accuracy of the force generated by the weight stack per se, to secure the unaffected transmission of the load without any friction from the guide rods through the plates of the weight stack. Based on the results showing that the actual load force on the shaft resembled the force level as calculated from the mass with a difference within −0.57 to 1.16% over the whole range, we are also confident that the weight stack is appropriate to be used for generating the force for the validation of forces measured by instrumented paddles. However, to secure an immediate transmission of load over time, it is recommended that the guide rods are kept well lubricated and carefully aligned to ensure minimal frictional resistance.

### 4.2. Validation of Instrumented Paddle Forces with the Orientation of Static Load Force That Is Orthogonal or Not Orthogonal Relative to the Face of the Blade

To investigate the applicability of using the jig for the validation of forces during static conditions with force direction applied either orthogonally or not orthogonally relative to the face of the blade, we used one brand of a commercially available instrumented paddle as an example. By comparing the reference measures of the jig with the measures from this paddle, we established validation examples for these conditions. With the force direction kept the same as that during calibration (orthogonal relative to the face of the blade), the values showed a deviation range of −3.4 to 3.0% with all values included (and 28 of 36 values within ±2%). When the force direction was changed by ±10 and ±20 degrees from the orthogonal direction relative to the face of the blade, a similar deviation range was observed (−2.9 to 4.1%). These results suggest that the peak force metrics are valid when measured with the specific instrumented paddle both with a force direction such as that during calibration and when rotated up to ±20 degrees. This was demonstrated with one specific brand of instrumented paddle only; however, the jig and the same procedures may be used with other brands as well. In a previous study [2], moveable sensor nodes mounted on a common competition paddle (Brasca IV, length 214 cm), were evaluated using a material testing machine (E3000, Instron, Norwood, MA, USA) to load the paddle blade together with a specialized rig for mechanical support at the handgrip positions. Evidence was presented that an average error of 0.4% can be achieved for blade force measurements up to 170 N directly after calibration and an average error of about 2% if the sensors were removed and re-attached between calibration and measurement. However, because of large differences in methods and procedures, our results are not simply comparable with this study or other previous studies in which the measured stroke forces have been validated by comparing applied static forces with measured forces. 

There are several benefits to being able to undertake precise static force measures with a jig. One is that, to the best of our knowledge, all commercially available instrumented paddles at the current time require calibration under standardized conditions prior to being used and for long-term follow-up on calibration stability. For example, large changes in temperature may affect both the sensor offset and gain factors and change the mechanical properties of the shaft. Also, it is unknown how the mechanical properties of a shaft may change over time due to regular use incorporating several thousands of repetitive bending cycles. Another benefit of being able to undertake precise static force measures with the jig is that it is easier to validate the force measures as they are read in the resulting output from the software that converts the sensor response values to force using the calibration information. 

Regarding appropriate force levels to be used for validation to cover the whole measuring range for both genders, it is important to take note of whether it is values for the hand or blade forces given since they differ due to the moment arm effect. In the literature, the information to establish these force levels is limited, especially with elite athletes performing maximal efforts during kayaking on water, where the highest forces are expected at the start of a race or in a short sprint. During a 200 m race at race pace in a K1, an average peak blade force of 274 N has been reported in a group of Portuguese elite male kayakers [16]. In 200 m at maximal effort in a K2, an average peak bottom hand force of around 344 N has been reported for elite male kayakers from the Singapore national team [8], and in a 10 s maximal sprint in a K1, an average peak blade force of approximately 450 N has been reported with a group of Swedish elite male kayakers [17]. Based on these results, we used a range of load force from 45 to 588 N (5–60 kg, with each plate of the weight stack weighing 5 kg) and we acknowledged that the bottom hand force is higher than the blade force due to the lever arm effect. A maximal force level of 588 N is likely above the need for most calibration and validation applications; however, since the maximal peak forces among the highest level of international male elite kayakers during maximal efforts is unknown, there may be a need to increase this range in the future. A maximal load of 800 or 900 N may then be needed to ensure the instrumented paddle characteristics are accurately assessed for male athletes with a reasonable measurement margin. If this is necessary, the current jig design may be modified to meet the requirement by adding additional 5 kg metal plates to the weight stack. Fortunately, this modification will also be well within the accurate range of the force sensors since the top hand and blade force sensors are certified for 2900 N and the bottom hand force sensor is certified for 19,600 N.

Further studies need to be conducted to determine the variation in the directions of forces in relation to shaft and blade positions during kayaking on water using the wing blade. However, since kinematic recordings of the blade path suggest that the blade is moving laterally away from the kayak [14], this indirect evidence indicates a variability in the direction of forces that warrants further investigation. Hence, if the direction of forces applied during on-water kayaking is not the same as the static conditions undertaken during calibration, it is beneficial that static force validation measures can be made using the current jig with the blade rotated to understand the effect of variations in the force directions. 

### 4.3. Validation of Force Measurements during Dynamic Conditions with Paddle Stroke Simulation

The design of a frame with superior stability in the current study permitted the application of kayak stroke simulations at high force levels and rates like kayaking in ecological conditions. Using the approach of comparing the reference measures with the jig and measures from an instrumented paddle, we established an example of the validation of paddle forces during dynamic force application, i.e., paddle-stroke simulation. With a target peak force of 400 N and paddle stroke simulations of 60 and 100 strokes/min, the common force variables were not significantly different between the instrumented paddle and the same measures by the jig. Hence, these results indicate that the specific instrumented paddle used is valid for recordings of stroke forces during on-water paddling at least under these conditions. In previous studies, little attention has been directed towards the equipment required for validation during dynamic conditions. As a minor part of one study [2], a material testing machine was programmed to apply a load on the paddle blade from 0 to 140 N within 0.1 s to create a realistic scenario like kayaking on water. With this approach, a delay in the rising slope and an overshoot was observed. In another, more recent study performed in closer-to-true ecological conditions [18], a strain gauge-equipped paddle shaft was used as a reference to evaluate the validity of paddle stroke key variables as measured by a commercially available instrumented paddle (Trainesense SmartPaddle). This study was performed with Finnish national-level canoe polo players in indoor swimming pools, and it was concluded that the SmartPaddle provides promising information on stroke key variables but needs further development before it can be used in everyday coaching sessions. 

Consequently, to the best of our knowledge, our study is the first to describe a jig capable of validating kayak stroke forces during dynamic conditions with forces and commonly calculated force metrics relevant for elite kayakers. In the current study, we demonstrated that peak forces up to about 600 N and stroke rates up to 100 strokes/min may be created manually using real-time feedback on a screen and exemplified common variables that can be calculated from the force curves such as peak force, time to peak force, impulse, and mean force for validation purposes. However, since kayaking on water during a K1 200 m race, with males at high international performance levels, involves higher stroke rates up to approximately 160–170 strokes/min and short stroke force times of about 300 ms, it is desirable that the response and accuracy of the instrumented paddles’ force sensors are evaluated under similar conditions in future studies. Likewise, in future studies, it is desirable that instrumented paddles’ force sensors are tested during conditions such as those produced by athletes in a K2 and K4 boat with even higher stroke rates and shorter stroke force times. 

### 4.4. Design and Practical Applications

With a commitment to extending the benefits of our work, we decided to describe the jig in a high level of detail that permits researchers, coaches, and equipment manufacturers of instrumented paddles to replicate the system and methods, and if desired, to build their own modifications and advancements. Building a frame using the alumina profiles resulted in making the jig stable and generally resistant to various small motions that may result in disturbances to the strain gauge signal. The need for a stable frame originates from our experiences of variation and tedious procedures existing using elementary stands similar to those described previously [19]. Specifically, the design and stability of the frame were feasible for enabling a common gym weight stack to be incorporated for the application of load. Using a weight stack with round steel bars through the weights minimizes the sway and enables an easy load application, either through the manual lifting of the metal blocks or by using an electrical winch as in our design. This aluminum profile system also offers several other advantages. For example, during the calibration of instrumented paddles, the profile open grooves enable the easy adjustment of the support devices to ensure differences in total paddle length and hand distances are accounted for. Also, during calibration procedures, it is crucial to have accurate horizontal measures at hand and blade positions since they are essential for the calculation of the force moments [7,19] and influence the accuracy of the lever arm effects. Hence, for this purpose and to align the paddle shaft between the top and bottom hand positions, the linear motion path with a display and sensors for the measurement of horizontal and vertical positions is useful. In addition, for further innovative developments, the jig design enables easy construction and the attachment of added devices, and from a practical point of view, it can be assembled and operated without professional skills in metal technologies and the availability of advanced mechanical workshops. 

In addition to undertaking the calibration and validation of force measures, as a safety measure, the mechanical strength of an instrumented paddle shaft may also be determined both during static and dynamic conditions with the jig. We are not aware of how manufacturers of ordinary paddle shafts undertake fatigue testing or mechanical strength tests, but the strength of ordinary shafts may be decreased by various changes to instrument them, such as adding sensors or adding/changing spigots, or simply by continual use and exposure to particular environments such as direct sunlight. Consequently, evaluating them in the jig after any structural modifications may verify if the mechanical strength is compromised. In addition, the design of the jig using a digital protractor, either attached to the rotatable device or the shaft, also permits the validation of angle measures that are included in some instrumented shafts by means of gyros in inertial measuring units. 

### 4.5. Limitations, Future Developments, and Future Studies

One limitation of the current jig version is the large size, heavy weight, and the time required to dismantle and assemble the frame, thereby making it less useful for mobile applications and better suited for stationary laboratory, factory or boat shed conditions. However, in future developments, the basic frame can potentially be constructed using an even lighter material, a reduced number of bracing connections, and another method of loading to replace the heavy weight stack. One alternative may be incorporating an electrical step motor that can be set to stop at different force levels for calibration and validation during static conditions. Ideally, it could be programmed to “mimic kayaking on water” with respect to stroke rates and applied forces, the time for stroke and air phase, time to peak force, etc.

Another limitation that likely may be conquered by future developments is that the current version requires some time-consuming manual data handling, i.e., it is not fully automated. For example, data collection and processing may be automated by custom written software with the automated down-sampling, filtering, and recognition of desired metrics both during static and dynamic conditions. In addition, it would be feasible with an option to include data from an instrumented paddle by using an easy import function and thereby obtaining a final report from a certain type of test such as force validation at static conditions or a test during dynamic conditions mimicking kayaking on water with output variables as mentioned above. It is also possible to find commercially available, less expensive force sensors, A–D interfaces, and software for data collection and processing or to reduce costs through in-house software development. In addition to these future developments, we hope the current work will result in future research on instrumented paddles as well as studies of shaft stiffness in which the linear motion path with a display and sensors for the measurement of horizontal and vertical positions would be useful to describe the bending characteristics of different paddles for sprint kayaking.

## 5. Conclusions

A novel jig with several new functions is presented that enables the calibration and validation of force measurements with instrumented paddles by providing standardized conditions for calibration and force validation during both static and dynamic conditions in a force range relevant to elite sprint kayaking.

## Figures and Tables

**Table 1:** Accuracy of jig force sensor measures.

	Mass (kg)	Force (N)	Jig Sensor Top Hand (N)	Diff. (%)	Jig Sensor Bottom Hand (N)	Diff. (%)	Jig Sensor Blade (N)	Diff. (%)
**A**	0.00	0.0	1.1		1.0		1.1	
	10.02	98.3	96.9	−1.43	99.3	1.10	98.5	0.29
	20.06	196.7	194.9	−0.90	198.9	1.09	197.3	0.28
	30.11	295.2	294.1	−0.39	297.3	0.71	296.7	0.51
	40.10	393.2	391.7	−0.38	396.1	0.73	394.7	0.39
	50.19	492.1	490.1	−0.41	495.3	0.65	494.1	0.39
	60.22	590.6	589.3	−0.20	594.8	0.71	593.3	0.46
**B**	60.22	590.6	588.5	−0.34	594.7	0.71	590.5	−0.01
	50.19	492.1	490.9	−0.24	496.0	0.78	495.4	0.65
	40.10	393.2	391.9	−0.33	396.7	0.89	395.0	0.46
	30.11	295.2	294.6	−0.20	298.4	1.08	297.5	0.76
	20.06	196.7	196.2	−0.26	199.6	1.46	197.9	0.57
	10.02	98.3	98.0	−0.28	100.0	1.76	98.4	0.17
	0.00	0.0	1.1		1.5		1.1	

**Table 2:** Accuracy of weight stack force.

Weight Stack		Jig Sensor Bottom Hand (N)	Diff. (%)
Mass (Kg)	Force (N)		
10.07	98.8	99.9	1.16
20.17	197.8	199.3	0.76
30.17	295.9	295.8	−0.02
40.22	394.4	393.0	−0.36
50.22	492.5	489.7	−0.57
60.22	590.6	590.7	0.02

**Table 3:** Accuracy of instrumented paddle force with force applied that is orthogonal relative to the face of the blade.

	Load		Top Hand Force (N)				Bottom Hand Force (N)				Blade Force (N)			
				Instr.	Diff.			Instr.	Diff.			Instr. Paddle	Diff.	
	(kg)	(N)	Jig	Paddle	(N)	(%)	Jig	Paddle	(N)	(%)	Jig	(BHF-THF)	(N)	(%)
**Left** **side**	10	98.3	35.8	36.2	0.5	1.3	97.8	100.2	2.3	2.4	63.6	63.9	0.3	0.5
	20	196.7	69.0	69.8	0.8	1.1	187.8	191.8	4.0	2.1	121.2	122.0	0.9	0.7
	30	295.2	108.1	109.1	1.0	0.9	293.5	300.7	7.1	2.4	188.8	191.6	2.8	1.5
	40	393.2	142.1	143.6	1.4	1.0	385.6	397.2	11.6	3.0	247.1	253.6	6.5	2.6
	50	492.1	179.7	181.7	2.0	1.1	488.3	498.1	9.8	2.0	312.5	316.4	3.9	1.2
	60	590.6	215.0	215.7	0.8	0.4	580.9	584.4	3.5	0.6	369.4	368.7	−0.8	−0.2
**Right** **side**	10	98.3	36.9	36.6	−0.2	−0.6	99.3	100.4	1.1	1.1	62.9	63.8	0.9	1.4
	20	196.7	73.1	72.9	−0.2	−0.2	196.4	201.2	4.8	2.4	125.2	128.3	3.1	2.5
	30	295.2	107.8	108.5	0.6	0.6	292.4	296.7	4.3	1.5	187.4	188.3	0.9	0.5
	40	393.2	142.9	143.5	0.6	0.4	386.0	392.8	6.8	1.8	246.0	249.3	3.3	1.3
	50	492.1	177.8	178.3	0.5	0.3	481.2	475.2	−6.1	−1.3	307.5	296.9	−10.6	−3.4
	60	590.6	216.8	216.2	−0.6	−0.3	580.8	582.8	1.9	0.3	367.6	366.6	−1.1	−0.3

**Table 4:** Accuracy of forces during force application ±10 and ±20 degrees from the orthogonal direction relative to the face of the blade.

Load			Force dir. +20°				Force dir. +10°				Orthogonal dir.				Force dir. −10°				Force dir. −20°		
			Jig	Paddle	Diff.		Jig	Paddle	Diff.		Jig	Paddle	Diff.		Jig	Paddle	Diff.		Jig	Paddle	Diff.
(kg)			(N)	(N)	(%)		(N)	(N)	(%)		(N)	(N)	(%)		(N)	(N)	(%)		(N)	(N)	(%)
10			98.1	99.5	1.5		99.3	102.0	2.7		98.1	98.3	0.2		97.6	98.8	1.2		98.3	100.0	1.7
20	**Bottom**		198.5	204.2	2.9		196.9	197.1	0.1		197.8	199.1	0.6		188.3	191.6	1.7		188.3	191.4	1.6
30	**Hand**		293.3	298.7	1.9		293.4	294.3	0.3		294.4	299.1	1.6		294.7	303.2	2.9		295.1	299.9	1.6
40	**Left**		386.6	394.8	2.1		384.4	381.2	−0.8		385.4	391.8	1.7		382.7	394.8	3.2		383.1	389.4	1.6
50	**side**		486.9	497.8	2.2		486.9	490.9	0.8		486.6	497.5	2.2		484.1	500.2	3.3		486.5	496.4	2.1
60			586.3	600.2	2.4		585.2	591.5	1.1		585.9	599.2	2.3		583.3	601.9	3.2		583.6	596.3	2.2
10			38.7	39.1	1.1		39.4	39.3	−0.1		38.9	38.5	−1.0		37.8	38.3	1.3		38.6	39.0	1.0
20	**Top**		79.2	79.1	−0.1		77.9	77.7	−0.2		78.1	77.4	−0.9		73.2	73.9	0.9		73.7	74.4	1.0
30	**Hand**		116.6	116.7	0.1		115.7	115.6	−0.1		116.4	115.6	−0.7		115.0	115.8	0.6		115.3	116.3	0.8
40	**Left**		153.3	153.8	0.4		150.8	151.1	0.2		152.1	151.4	−0.4		150.0	150.8	0.5		149.6	150.8	0.8
50	**side**		193.4	193.9	0.3		191.8	192.1	0.2		191.7	191.5	−0.1		189.9	190.9	0.5		189.3	191.5	1.2
60			233.0	233.9	0.4		231.0	231.3	0.1		230.7	230.9	0.1		228.4	230.0	0.7		226.9	230.2	1.4
10			97.5	101.5	4.1		98.0	98.0	0.0		99.2	96.4	-2.8		98.9	100.4	1.5		99.4	100.8	1.4
20	**Bottom**		197.2	205.0	3.9		197.4	200.6	1.6		188.9	188.7	-0.1		197.6	202.9	2.7		197.1	202.7	2.9
30	**Hand**		293.5	304.3	3.7		293.4	299.7	2.1		289.3	292.5	1.1		293.0	300.1	2.4		289.2	298.2	3.1
40	**Right**		382.6	397.0	3.8		383.3	392.8	2.5		385.0	391.8	1.8		386.4	397.3	2.8		386.3	399.2	3.3
50	**side**		484.9	503.5	3.8		484.7	497.6	2.7		485.4	497.4	2.5		486.6	501.5	3.1		486.6	505.7	3.9
60			584.8	607.9	4.0		584.4	600.1	2.7		584.7	601.6	2.9		586.4	603.9	3.0		586.6	610.0	4.0
10			39.5	40.0	1.4		39.6	38.7	−2.2		39.9	38.8	−2.9		39.6	39.4	−0.6		38.8	38.7	−0.3
20	**Top**		79.0	79.8	1.0		79.0	78.1	−1.1		75.4	73.9	−2.1		78.2	77.7	−0.7		77.7	76.9	−1.0
30	**Hand**		116.3	117.9	1.4		116.8	116.0	−0.7		114.7	113.1	−1.4		114.9	114.5	−0.4		113.0	112.7	−0.3
40	**Right**		150.8	153.4	1.7		152.3	151.7	−0.4		152.2	150.7	−1.0		151.6	151.2	−0.2		150.9	150.7	−0.2
50	**side**		190.8	194.3	1.8		191.9	191.9	0.0		191.7	190.5	−0.6		190.7	190.7	0.0		189.4	190.5	0.6
60			230.3	234.6	1.9		230.8	231.2	0.2		231.3	230.0	−0.6		229.5	229.7	0.1		228.2	229.8	0.7

## Data Availability

Data are contained within the article.

## Appendix A

**Table A1:** Accuracy of instrumented paddle force metrics during dynamic force application on left side with 400 N target peak force.

		Impulse Duration (S)		Peak Force (N)		Time to Peak Force (s)		Mean Force (N)		Force Impulse (Ns)	
	TCnr	Instr. Paddle	Jig	Instr. Paddle	Jig	Instr. Paddle	Jig	Instr. Paddle	Jig	Instr. Paddle	Jig
	1	0.48	0.48	341.4	339.7	0.20	0.22	165.6	166.1	79.5	79.7
	2	0.51	0.51	431.9	431.9	0.24	0.24	209.3	206	106.7	105.1
	2	0.53	0.53	457.9	449.9	0.26	0.27	201.3	200.1	106.7	106.1
60	4	0.53	0.53	393.7	386.0	0.26	0.28	178.2	178.6	94.4	94.7
Strokes	5	0.54	0.54	383.0	375.3	0.25	0.26	185.1	185.7	100.0	100.3
per min	6	0.49	0.49	413.6	410.9	0.22	0.22	204.9	205.7	100.4	100.8
	7	0.57	0.57	394.4	386.0	0.27	0.30	182.3	182.8	103.9	104.2
	8	0.54	0.54	431.4	423.9	0.25	0.27	202.7	204.4	109.5	110.4
	8	0.52	0.52	416.5	410.2	0.24	0.25	206.5	205.2	107.4	106.7
	10	0.49	0.49	406.1	397.6	0.24	0.25	205.6	210.7	100.7	103.2
	**Mean**	**0.52**	**0.52**	**406.99**	**401.1**	**0.24**	**0.26**	**194.2**	**194.5**	**100.9**	**101.1**
	**SD**	**0.03**	**0.03**	**31.90**	**31.5**	**0.02**	**0.03**	**15.1**	**15.0**	**8.8**	**8.6**
	1	0.44	0.44	406.0	400.7	0.19	0.19	206.1	202.4	90.7	89.1
	2	0.40	0.40	409.8	404.7	0.15	0.17	218.5	216.4	87.4	86.6
	2	0.41	0.41	415.8	409.2	0.16	0.17	213.2	212.8	87.4	87.2
100	4	0.43	0.43	418.7	412.5	0.18	0.18	205.8	206.8	88.5	88.9
Strokes	5	0.41	0.41	397.9	397.1	0.17	0.17	205.3	205.7	84.2	84.3
per min	6	0.43	0.43	389.7	386.0	0.18	0.19	198.5	199.6	85.4	85.8
	7	0.44	0.44	435.0	427.3	0.16	0.16	230.0	226.7	101.2	99.7
	8	0.40	0.40	427.5	423.1	0.17	0.17	222.5	221.7	89.0	88.7
	8	0.41	0.41	374.4	372.7	0.17	0.18	187.7	189.4	77.0	77.7
	10	0.49	0.49	394.4	385.4	0.21	0.22	239.9	238.6	117.6	116.9
	**Mean**	**0.43**	**0.43**	**406.9**	**401.9**	**0.17**	**0.18**	**212.8**	**212.0**	**90.8**	**90.5**
	**SD**	**0.03**	**0.03**	**18.4**	**17.2**	**0.02**	**0.02**	**15.4**	**14.4**	**11.2**	**10.8**
